# Electrogram Dynamics at the Site of Ventricular Tachycardia Termination During Radiofrequency Ablation

**DOI:** 10.19102/icrm.2025.16071

**Published:** 2025-07-15

**Authors:** Wissam Harmouch, Ali Saad Al-Shammari, Muhie Dean Sabayon, Arun Narayanan, Haider Al Taii

**Affiliations:** 1 Department of Internal Medicine, University of Texas Medical Branch, Galveston, TX, USA; 2 Department of Internal Medicine, College of Medicine, University of Baghdad, Baghdad, Iraq; 3 Department of Cardiovascular Medicine, University of Texas Medical Branch, Galveston, TX, USA

**Keywords:** Electrogram, mapping, radiofrequency ablation, structural heart disease, ventricular tachycardia

## Abstract

Ventricular tachycardia (VT) is a life-threatening arrhythmia associated with high morbidity and mortality, particularly in patients with structural heart disease. Radiofrequency ablation is an effective procedure to treat patients with this malignant arrhythmia. We report three cases of successful termination of VT using unique catheter ablation techniques. Through these cases and techniques, we highlight the advantages of specific localization of abnormal circuits within the cardiac layer involved, as well as electrogram evidence of tachycardia termination.

## Introduction

Ventricular tachycardia (VT) is a life-threatening arrhythmia associated with high morbidity and mortality, particularly in patients with structural heart disease (SHD), often necessitating catheter ablation for effective management.^1^ Radiofrequency ablation (RFA) is critical in treating VT, especially among patients with SHD. This minimally invasive technique uses a high-frequency alternating current to ablate targeted cardiac tissues, effectively addressing arrhythmias such as VT.^2^ By combining various mapping techniques during RFA, procedural effectiveness can be enhanced, and the recurrence of arrhythmias can be reduced, achieving acute success rates of 85% and a VT-free survival rate of 68.5% over a median follow-up period of 25.5 months.^3^

Electrogram (EGM) signals are critical in guiding the ablation of VT, providing essential insights into the location of the abnormal electrical activity. Accurate detection of low-voltage late potentials within the scar tissue is crucial, as these signals often mark regions of interest for ablation. Advanced recording systems that improve signal clarity enhance the precision of identifying these abnormal signals, ensuring more effective ablation therapy. Recent advances in mapping strategies, such as eliminating local abnormal ventricular activities, underscore the importance of accurately identifying abnormal EGMs within the scar tissue. These EGMs represent local electrical activity and provide vital information about the arrhythmogenic substrate, particularly in regions with altered conduction properties that sustain the VT circuit.^4^

Integrating multi-catheter systems allows for more precise mapping of critical arrhythmia circuits, employing techniques such as activation mapping, entrainment mapping, pace mapping, and substrate mapping. This has led to a reduction in procedural times and improved success rates.^5^ Furthermore, novel mapping technologies enable the reconstruction of three-dimensional (3D) cardiac models by combining electrical and anatomic data, thereby minimizing reliance on fluoroscopy.^6^

This case series underscores the significance of EGM analysis in managing VT by demonstrating sequential EGMs during RFA and the outcome of termination of tachycardia.

## Case 1

**Figure 1:**
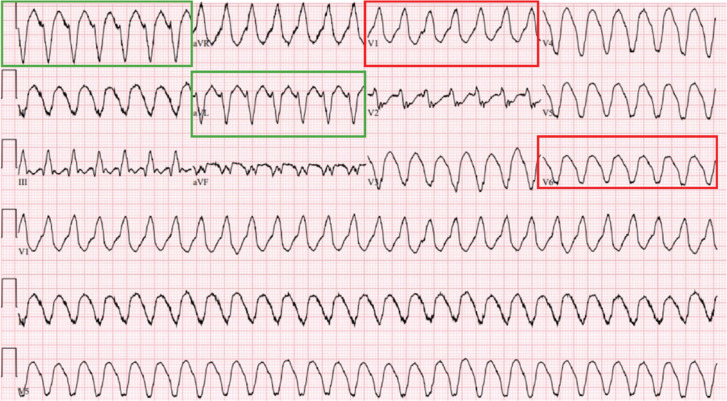
Presenting electrocardiogram in case 1. Monomorphic ventricular tachycardia originating from the left circumflex territory suggested by the right bundle branch block morphology in V1 with negative vectors in V6, suggesting an origin from the left ventricular apex (red outline). The negative vectors in lead I and aVL suggest an origin from the apical lateral wall (green outline).

A 59-year-old man with a history of atrial fibrillation, coronary artery disease with percutaneous coronary intervention (PCI) of the left circumflex (13 years prior), chronic obstructive lung disease (COPD), obstructive sleep apnea (OSA), and heart failure with reduced ejection fraction (HFrEF) of 35%–40% presented with chest pain and palpitations while laying down. An electrocardiogram (ECG) showed monomorphic VT at a rate of 200 bpm **(Figure 1)**. Despite treatment with intravenous amiodarone, the patient continued to be symptomatic. Defibrillation at 200 J was given, and the patient returned to normal sinus rhythm. An echocardiogram revealed an ejection fraction (EF) of 35%–40%, grade 3 diastolic dysfunction, normal left ventricular (LV) wall thickness, and hypokinetic inferior and inferolateral walls from the base to mid-cavity, as well as hypokinetic apical inferior and apical lateral walls. There was no significant valvular disease. The patient was stabilized in the cardiac care unit and initially was not amenable to interventions but ultimately decided to pursue catheterization. Approximately 72 h after presentation, he underwent a coronary angiogram, which revealed a chronic total occlusion in the left circumflex artery and a 70%–80% stenosis in the proximal to mid-right coronary artery that was amenable to PCI. The patient was clinically stable after catheterization and ultimately underwent implantation of an implantable cardioverter-defibrillator (ICD) for the prevention of sudden cardiac death. Upon discharge, he was prescribed an oral amiodarone taper.

Six weeks after discharge, he presented for VT ablation. The patient was prepped and draped accordingly using contemporary methods. Using intracardiac echocardiography (ICE) and fluoroscopy guidance, the EPstar catheter (Boston Scientific, Marlborough, MA, USA) was placed in the lateral ventricular branch of the coronary sinus to map the epicardial surface of the scar. Through venous access and eventual transseptal puncture, the Orion high-density mapping catheter (Boston Scientific) was used to create a 3D electroanatomic map of the right ventricle and LV during tachycardia using the Rhythmia mapping system (Boston Scientific). The mapping settings used were a 30-Hz high-pass filter and a 300-Hz low-pass filter. Small boluses of heparin were administered during the procedure to maintain an activated clotting time (ACT) range of 300–400 s. The zone of slow conduction was localized to the LV area between the apex and mid-cavity of the lateral inferior segment, which correlated with the origin of the VT visualized from the ECG **(Figure 1)**.

**Figure 2:**
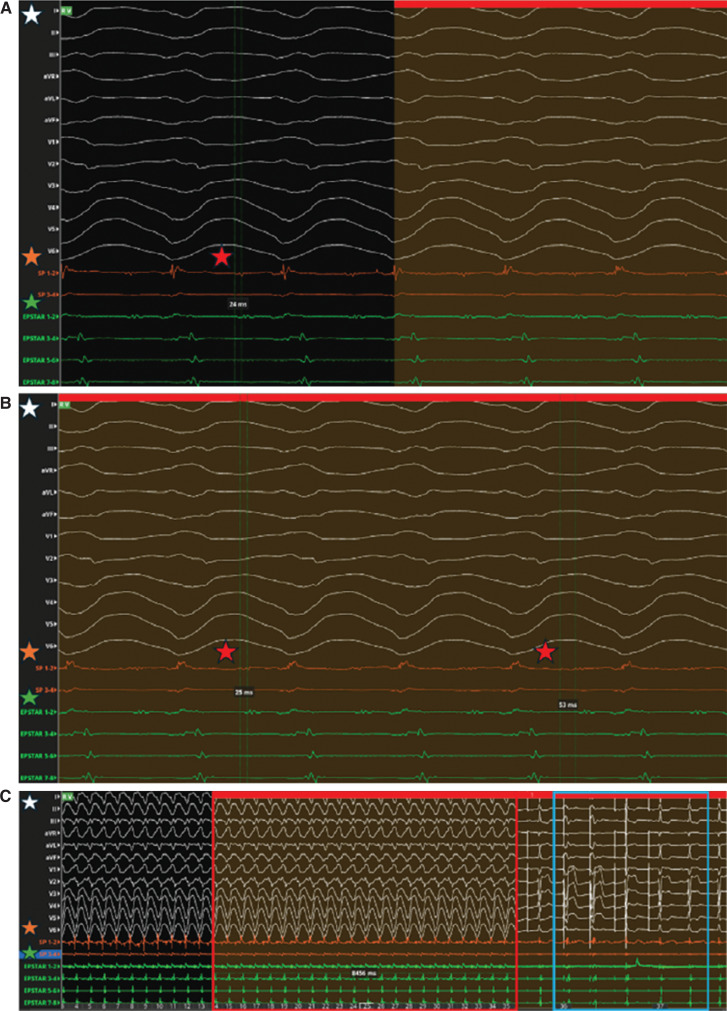

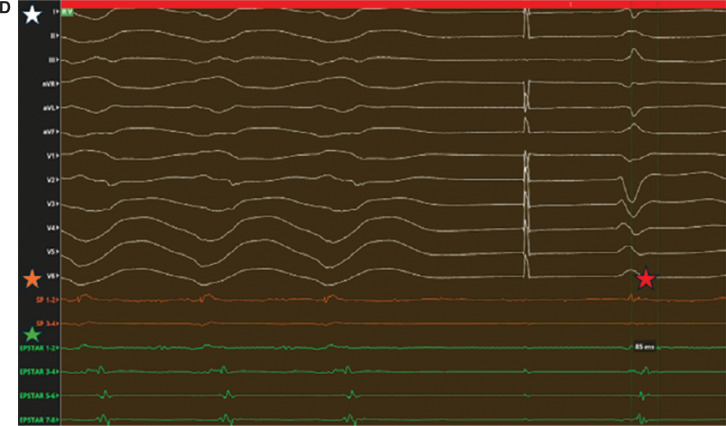
Electrocardiogram during case 1. **(A)** Prior to ablation, the electrogram (EGM) revealed a mid-diastolic signal splitting, 24 ms in duration (red star), during ventricular tachycardia that represented slow conduction as the origin of the tachycardia. **(B)** With ablation, the duration of the signal splitting increased from 25 to 53 ms (red stars). **(C)** After applying ablation lesions (EGM outlined in red), the tachycardia terminated. Despite pacing, tachycardia was not induced (EGM outlined in blue). **(D)** A final EGM revealed no more ventricular tachycardia after completion of the ablations, with the mid-diastolic signal duration noted to be 85 ms (red star). The white star represents the electrocardiogram, the orange star represents the EGM generated by the Orion catheter in the left ventricle, and the green star represents the EGM generated by the EPstar catheter in the coronary sinus.

Specifically, the local EGM revealed a mid-diastolic signal splitting that measured 24 ms in duration **(Figure 2A)**. An RFA lesion at 35–40 W for long durations was given, which yielded a longer duration between the signal splitting, ultimately 53 ms **(Figure 2B)**, with eventual termination of the VT **(Figure 2C)**. The local EGM revealed the low-voltage mid-diastolic signal to be measured at 85 ms at the termination site **(Figure 2D)**. After ablation, isoproterenol and stimulation pacing were used to induce VT; however, they did not induce any VT. The patient did well post-ablation, with no more episodes of sustained VT clinically or electrocardiographically on interrogation of the ICD. One month after the ablation, the patient followed up in the clinic without any presyncope symptoms, chest pain, palpitations, or other signs of recurrent VT. At his 4-month follow-up, the patient continued to report that he was doing well. An interrogation of his ICD at 4 months revealed no runs of VT.

## Case 2

A 67-year-old man with a history of four-vessel coronary artery bypass (12 years prior), COPD, OSA, abdominal aortic aneurysm with endovascular aneurysm repair (9 years prior), and HFrEF of 10%–15% with a cardiac resynchronization therapy-defibrillator (CRT-D) device (via His pacing) was admitted after his CRT-D delivered two shocks without any prodromal symptoms of presyncope, syncope, or palpitations. An echocardiogram revealed a severely dilated LV with an EF of 10%–15%. Multiple wall motion abnormalities were noted, including aneurysmal apical septal and lateral walls, as well as an aneurysmal apex. The mid-anteroseptal and apical anterior and inferior walls were akinetic, with the remaining walls noted to be hypokinetic. There was no clinically significant valvular disease. During the first 48 h of admission, the patient had frequent non-sustained VT on telemetry and was started on intravenous amiodarone. Four days after the presentation, he underwent VT ablation for recurrent symptomatic monomorphic VT in the setting of prior ischemia.

After prepping and draping the patient accordingly, venous access was achieved under ultrasound guidance. Using ICE and fluoroscopy, the EPstar catheter (Boston Scientific) was placed in the anterior interventricular vein distal to the coronary sinus, and the Trideca catheter (Access Point, Minneapolis, MN, USA) was placed in the LV after successful transseptal puncture. A 3D electroanatomic map of the LV was created during LV pacing using the EnSite mapping system (Abbott, Chicago, IL, USA). The mapping settings used were a 30-Hz high-pass filter and a 300-Hz low-pass filter. An ACT range of 300–400 s was maintained throughout the procedure.

**Figure 3:**
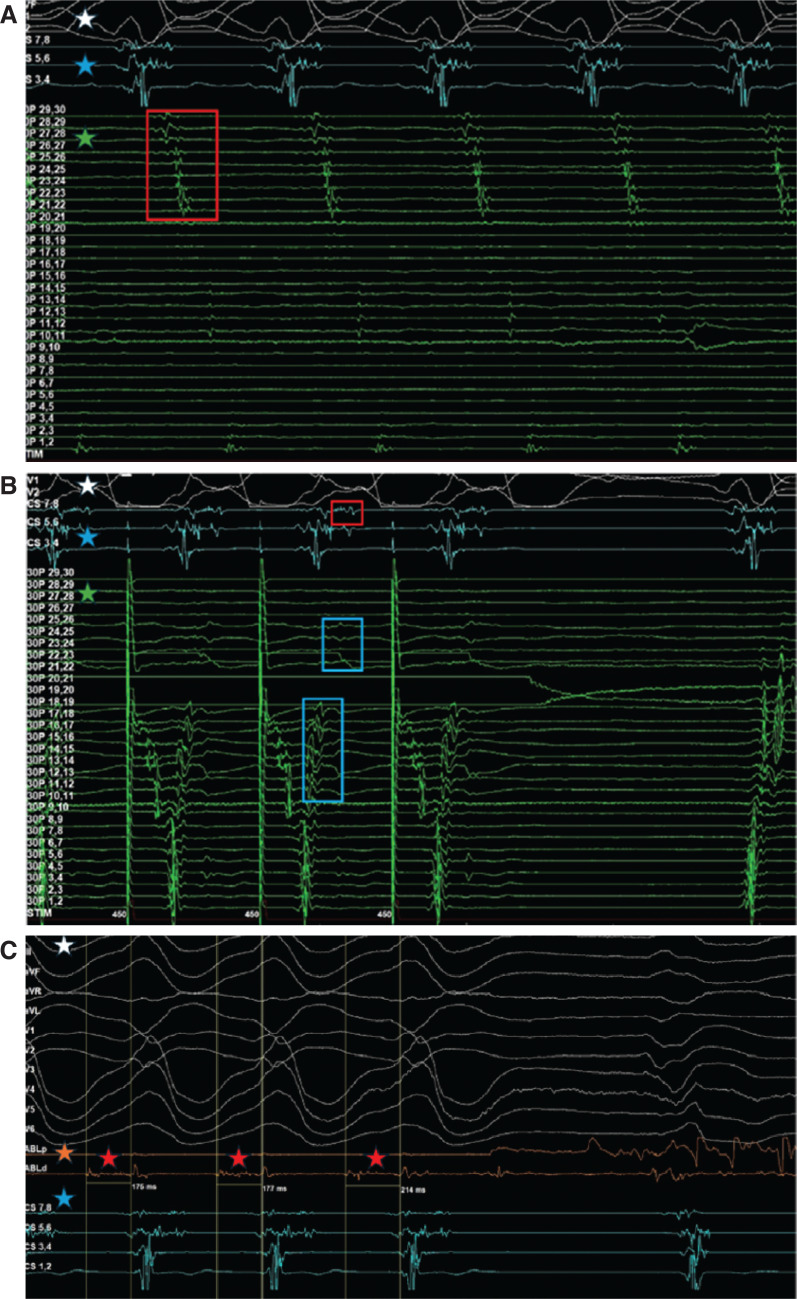
Electrograms (EGMs) recorded during case 2. **(A)** Multiple areas of mid-diastolic potentials were identified on the EGM using the Trideca catheter (EGMs outlined in red), as the ventricular tachycardia foci were mapped to the left ventricular (LV) anterior lateral wall. **(B)** Pacing from the Trideca catheter at points 20, 21 at 450 ms revealed a second zone of slow conduction in the LV mid-inferior and basal inferoseptal walls corresponding to EPstar points 7, 8 (EGM outlined in red). Also, late low-amplitude diastolic signals were noted from Trideca points 11–18 that occurred closer to pacing points 20, 21 with a line of block noted at points 21, 22 (EGMs outlined in blue). **(C)** After multiple ablation lesions were applied, the tachycardia was terminated as displayed by the white EGMs. The mid-diastolic signal was initially 175 ms in duration and prolonged with each ablation applied from 177 ms to ultimately 214 ms (red star). The white star represents the electrocardiogram, the blue star represents the EGM generated by the EPstar catheter in the coronary sinus, the green star represents the EGM generated by the Trideca catheter in the LV, and the orange star represents the EGM generated by the Orion catheter inside the LV.

After pacing at 450 ms, the zone of slow conduction was localized to the LV apical septal–lateral and inferior walls. The extent of slow conduction with multiple areas of mid-diastolic potentials was identified on the EGM **(Figure 3A)**. Pacing from these areas did not show concealed fusion. We also noted that pacing from these different areas terminated the VT after multiple capture beats. At the anterior lateral wall, the VT terminated during early beats of overdrive pacing. While initially mapping, a second zone of slow conduction was noted in the LV mid-inferior and basal inferoseptal walls. After pacing the Trideca catheter at points 20 and 21 at 450 ms, an area of slow conduction with low amplitude was noted on the EGM corresponding to EPstar points 7 and 8 **(Figure 3B)**. This suggested an intramural scar as the etiology of the VT. Multiple RFA lesions were applied to that area with subsequent termination of the tachycardia **(Figure 3C)**. Isoproterenol and pacing stimulation did not induce VT. The patient was started on intravenous amiodarone and did not have any more episodes of VT. Five days after the procedure, the patient developed hospital-acquired pneumonia and subsequent acute hypoxic respiratory failure requiring intubation and mechanical ventilation. Continuous telemetry did not reveal any arrhythmias, and an echocardiogram did not show any new functional or structural abnormalities. Ultimately, 6 days after the procedure, the patient passed away from progressive hypoxemia from pneumonia.

## Case 3

A 66-year-old man with a history of non-ischemic HFrEF of 35%–40% following ICD implantation (with coronary angiography 1 week prior showing patent coronary arteries), seizure disorder, and remote history of outside of hospital cardiac arrest presumed secondary to VT presented to the hospital due to dizziness and light-headedness followed by his ICD firing. Device interrogation showed multiple episodes of VT that resolved with anti-tachycardia pacing. However, interrogation also revealed an episode of VT that persisted despite anti-tachycardia pacing and triggered a defibrillation, which ultimately terminated the VT. Upon presentation, he was on metoprolol succinate 100 mg daily and amiodarone 200 mg daily. An echocardiogram was obtained and showed an EF of 35%–40% with hypokinetic walls from base to mid-cavity, which was unchanged from 1 week prior. He was taken for an electrophysiology study and VT ablation.

After prepping and draping the patient accordingly, venous access was achieved under ultrasound guidance. Using ICE and fluoroscopic guidance, the EPstar catheter (Boston Scientific) was positioned in the distal tip of the coronary sinus in the anterior interventricular vein. Prior to transeptal puncture, ICE was used to examine the structures of the right ventricular inflow tract, LV inflow tract, and adjacent structures and remained in the right ventricle to monitor for any potential procedural complications. Next, a Baylis transeptal needle wire (Boston Scientific) was used to perform transeptal puncture. The Advisor™ HD Grid mapping catheter (Abbott) was introduced retrograde into the LV, and 3D electroanatomic mapping of the LV was performed.

**Figure 4:**
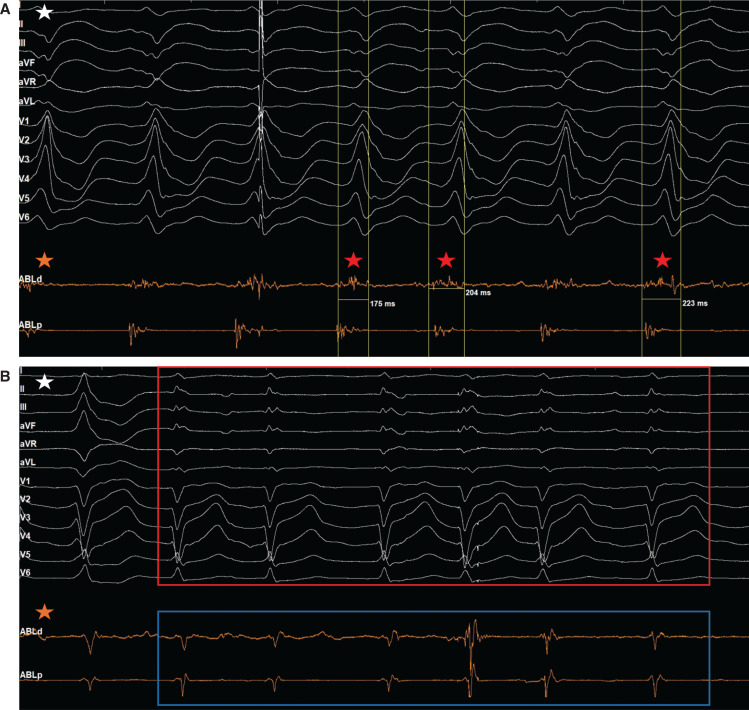
Electrograms (EGMs) taken during Case 3. **A:** The EGM reveals near-field pre-systolic signals on the distal end of the TactiFlex™ catheter (APLd) that corresponded to the ventricular tachycardia exit. During radiofrequency ablation, the duration of the pre-systolic signal was noted to prolong in duration from 175 ms (red star) to 204 ms (red star) and ultimately to 223 ms (red star). **B:** Post-ablation, the EGM revealed normal signals at APLd (blue outline) corresponding to normal QRS morphologies on the electrocardiogram (red outline), indicating that the ventricular tachycardia was terminated. White star indicates the electrocardiogram. Orange Star indicates the EGM generated by the TactiFlex™ catheter inside the left ventricle.

Using stimulation pacing, areas of abnormal conduction were noted along the basal posterior septal walls. However, full cycle lengths were not able to be obtained. This suggested that the isthmus was partly intramural, and that the abnormal conduction was the VT exit. The Advisor™ HD Grid mapping catheter was removed and a TactiFlex™ ablation catheter (Abbott) was introduced into the LV. The distal end of the TactiFlex™ catheter (APLd) showed fractionated far-field mid-diastolic signals with near-field pre-systolic signals. These pre-systolic signals corresponded to the VT exit where RFA lesions were applied. With RFA, the duration of the pre-systolic signal was noted to increase from 175 ms initially to 204 ms and then ultimately to 223 ms **(Figure 4A)**. Post-ablation, normal signals were noted at APLd corresponding to normal QRS morphologies, indicating that the VT was terminated **(Figure 4B)**. Post-ablation, stimulation pacing was used to induce VT; however, there was no VT noted. The patient did well post-procedure without any more episodes of VT and was discharged with metoprolol succinate 100 mg daily.

## Discussion

Our three cases of VT managed through catheter ablation showed promising outcomes. Each case highlights the distinct aspects of VT dynamics and effective ablation techniques. In the first case, the local EGM measured before and after RFA provided critical insight into electrophysiological changes associated with successful VT termination.

Prior to RFA, the local EGM revealed the duration of slow conduction to be 24 ms, which indicated strong electrical signals at the termination site. This suggested that this location was closely associated with the VT circuit. This proxy makes it a prime target for ablation, as it reflects the early capture of the electrical impulse that sustains the arrhythmia.

The successful termination of VT during or immediately after the ablation serves as a key indicator. This event confirms that the abnormal re-entrant circuit responsible for the VT has been effectively interrupted. This aligns with findings from the existing literature showing similar outcomes, with an emphasis on the importance of timely intervention of VT management.^1,2^ Furthermore, the subsequent measurement of 85 ms at the same site indicates a significant alteration in local electrical activity following VT termination. This delay suggests that the re-entrant circuit has been modified or eliminated, consistent with post-ablation expectations where normal conduction pathways are restored.^3,4^

Our second case further illustrates the complexities of identifying and targeting VT circuits using advanced mapping techniques. Using the Trideca catheter was of paramount importance as it has 30 electrodes that are spaced 1 mm apart. This allows for understanding the finer details of the abnormal circuit. The detection of mid-diastolic signals on the Trideca catheter positioned in the LV underscores the presence of critical slow conduction pathways. These pathways are often implicated in sustained VT and indicate the importance of precise mapping in identifying potential ablation targets. Pacing at 450 ms with delays witnessed on the EGM corresponding to the epicardial catheter indicated an area of slow conduction. This finding suggested a possible target for intervention. Similar pacing strategies have been used in the literature and advocate for pacing strategies during ablation procedures.^5,6^ The precise localization of the VT origin as highlighted by case 2 allows for efficiency in ablation, by preserving healthy myocardium. This preservation is even more important in patients with reduced EF. In addition to pacing strategies, using multielectrode mapping catheters has also been discussed in the literature as an alternative mapping technique in VT. Specifically, Hadjis et al. revealed that using multielectrode catheters during activation mapping to visualize the complete diastolic pathway was associated with a reduced incidence of VT recurrence.^7^ Furthermore, advancements in imaging, such as cardiac computed tomography and cardiac magnetic resonance, have emerged as tools for a better understanding of scar burden, fibrosis, and the underlying substrate for VT.^8^

EGMs are vital for identifying successful termination of VT, but they also have a paramount role in identifying scar location and relation to the circuit. This was highlighted in Case 3. Specifically, the distinctive sharp voltage mid-diastolic potentials that were seen in Cases 1 and 2 reflected the isthmus in their respective circuits with transmural and epicardial involvement, respectively, whereas, in Case 3, the near-field pre-systolic signals noted on APLd corresponded to the VT exit and intramural involvement of the circuit. Moreover, Cases 1 and 2 involved ischemic scars, whereas Case 3 involved a non-ischemic scar. Ischemic scars are known to involve the endocardium and can have transmural involvement depending on the ischemia severity. On the other hand, non-ischemic scars are not as well understood and can involve any layer of the myocardium. Simply, the EGM morphology and location within the VT cycle allows electrophysiologists to understand the scar location and relation to the VT circuit.

These cases demonstrate that careful analysis of EGM signals before, during, and after ablation can significantly enhance the understanding of the VT mechanism and improve procedural outcomes. Integrating advanced mapping techniques and real-time monitoring helps identify effective targets for intervention and supports the ongoing advancement in catheter-based therapies for VT management. Further studies and robust research should continue to explore these advancements, refine treatment protocols, and improve patient care strategies for VT.
